# Detection of Microstructural Changes in Metastable AISI 347, HSS Z-M4 and Tool Steel Ferrotitanit WFN by Mechanical Loss Coefficient at Ultrasonic Frequencies

**DOI:** 10.3390/ma15176002

**Published:** 2022-08-30

**Authors:** Moritz Liesegang, Tobias Daniel, Benedikt Jäckels, Marek Smaga, Tilmann Beck

**Affiliations:** Institute of Materials Science and Engineering, Technische Universität Kaiserslautern, 67653 Kaiserslautern, Germany

**Keywords:** ultrasonic processes, sonotrodes, modal analysis, loss coefficient, phase transformation, cyclic load, VHCF

## Abstract

Ultrasonic processes such as ultrasonic welding or ultrasonic fatigue testing use power ultrasound to stimulate materials with amplitudes in the range of 1–100 µm. The ultrasonic welding process is sensitive to any changes in the system or even the environment that may result in lower joint quality. The welding tools, so called sonotrodes, have to be accurately designed to endure high mechanical and thermal loads while inducing a sufficient amount of welding energy into the joining zone by oscillation with the Eigenfrequency of the whole system. Such sonotrodes are often made of thermally treated metals where the heat treatment is accompanied by microstructural changes. During ultrasonic stimulation, the material may further change its properties and microstructure due to cyclic loading. Both are expected to be recognized and identified by loss coefficients. Therefore, the loss coefficient was determined by modal analysis of rods and fatigue specimen made of different materials to correlate microstructural changes to attenuation. The determined loss coefficients indicated microstructural changes in all materials investigated, confirming results from previous investigations that showed an increasing attenuation due to cyclic loading for AISI 347. For the sonotrode materials Z-M4 PM and Ferrotitanit WFN, the loss coefficients decreased due to thermal treatments. Technically most relevant, changes in elastic modulus due to thermal treatments were quantitatively related to frequency changes, which can significantly simplify future sonotrode development.

## 1. Introduction

Ultrasonic welding (USW) is based on a high frequency oscillation of the sonotrodes to achieve firmly bonded joints. High performance sonotrodes e.g., for the welding of titanium to carbon fibre reinforced plastics (CFRP) [1] require a high displacement amplitude at the sonotrode tip to induce a sufficient amount of energy into the joining zone. Such high amplitudes come along with high mechanical stress amplitudes in the sonotrodes that may cause sudden failure [2]. A prediction of failure in high performance sonotrodes that operate close to their fatigue limit is essential for industrial ultrasonic welding lines to assure high product quality at acceptable costs. Material, sonotrode geometry, and sonotrode tip topography have to be designed for successful ultrasonic welding in general, considering that the operation frequency of the machine has to be equal to the Eigenfrequency of the desired modal shape of the sonotrodes. Most powder metallurgical high performance metals change their acoustic behaviour, especially their Eigenfrequency, by the required thermal treatment after machining, which complicates the sonotrode design significantly [2].

Attenuation has been previously investigated in correlation to microstructural changes for various materials. When considering the microstructure, it was noted that attenuation is influenced by the dislocation density, which increases during cyclic loading [3]. According to this, the lattice is more disturbed at high dislocation density and the propagation of vibrations in the crystal is made more difficult. It is described that, in addition to the dislocation structure, other microstructural changes e.g., stacking faults, twins, phase transformation, pores or microcracks can also be responsible for a stronger attenuation [4,5]. Some specific investigations of metals about attenuation behaviour, evaluated by loss coefficient, are listed in Table 1. Additionally, grain size and shape that possibly change by thermal treatment, may affect the acoustic properties of the material [6,7].

The approach of using the mechanical loss coefficients to detect microstructural changes presented in this study faces both thermally and deformation induced austenite-martensite transformation. The loss coefficient is expected to be very sensitive to any alterations in materials and components and hence is suitable to detect even small changes, which is valuable for quality control and process monitoring by non-destructive and rapid modal analysis [2].

## 2. Materials and Methods

### 2.1. Materials

The performance of sonotrodes is mainly determined by mechanical and acoustic properties as well as the wear behaviour of the materials. Tensile strength and hardness are considered as key mechanical properties, while Young’s modulus, Poisson’s ratio and mass density are necessary to calculate the dynamic behaviour during oscillation [1,2,10].

Z-M4 PM, a high-speed tool steel, typically used for metal drilling was the first sonotrode material investigated. Despite its lower wear resistance in comparison to the similar, commercially used sonotrode tool steel CPM 10 V, according to the manufacturer’s data sheet [11], Z-M4 is an interesting material for sonotrodes because of its relatively higher impact strength [12] and hence, an increased damage tolerance. The second investigated sonotrode material Ferrotitanit WFN is commercially used as a sonotrode material. It consists of a martensitic matrix, reinforced by approximately 33 wt% titanium carbide that makes it one of the hardest but still machinable materials [13]. In comparison to other sonotrode materials, the wear resistance of WFN seems to be higher [14].

The chemical compositions of both sonotrode materials investigated are listed in Table 2 and Table 3.

The metastable austenitic stainless steel AISI 347 was the third material investigated as it is prone to deformation induced phase transformations from austenite to α′-martensite under cyclic loading. In contrast to the sonotrode materials that are investigated before and after a thermal treatment, the microstructural changes in AISI 347 were observed quasi continuously during the fatigue process. Table 4 shows the chemical composition, determined by spectral analysis, of the investigated AISI 347 batch. The chemical composition significantly influences the metastability of austenitic stainless steels, which can be characterized by the stacking fault energy (SFE) or martensite deformation temperature M_d30_. The SFE of the investigated AISI 347 is 42 mJ/m^2^ and M_d30_ = 25 °C [15].

All three materials investigated face an austenite-α′-martensite phase transformation. For the sonotrode materials the transformation is driven by the thermal treatment that is required to achieve the requested technical properties, especially a high hardness [1,2,10,16]. Because the investigated AISI 347 is metastable at ambient temperatures, the deformation induced austenite-α′-martensite transformation occurs due to cyclic loading. Despite the fact that the driving forces for the phase transformations are different, the loss coefficient is expected to decrease for all specimens as shown in similar previous studies [17].

**Figure 1:**
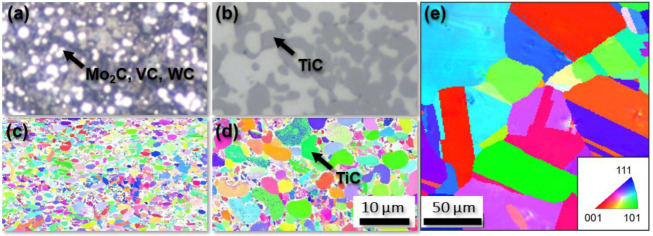
Microstructure including reinforcing phases of Z-M4 (**a**,**c**), WFN (**b**,**d**) and AISI 347 (**e**) observed by light optical microscopy (**a**,**b**) and by EBSD (**c**–**e**).

Figure 1 shows the microstructure of all three investigated materials Z-M4 (Figure 1a,c), WFN (Figure 1b,d) and AISI 347 (Figure 1e) determined by light optical microscopy (a,b) and electron backscatter diffraction (EBSD) (Figure 1c–e). The colours used in the EBSD micrographs indicate the texture of the material but were used only for improved visibility of the grain structure in this case. The line-intercept method was used to determine grain sizes and digital image analysis was used to determine the amount of reinforcing phase considering the proportionate area of each phase. These microstructural characteristics are listed in Table 5.

**Figure 2:**
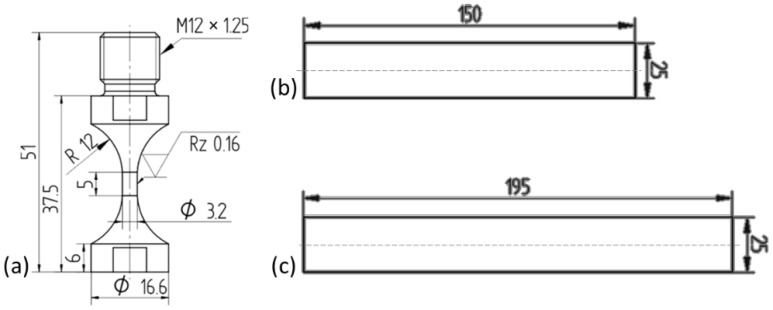
Geometries of (**a**) the VHCF-specimen AISI 347 and the rods (**b**) Z-M4 and (**c**) WFN. (Unit: mm).

The geometry of the AISI 347 VHCF-specimen and the investigated rods made from the sonotrode materials Z-M4 and WFN is shown in Figure 2. The comparatively complex geometry of the VHCF-specimen is required for ultrasonic fatigue testing while the rods were chosen to minimise the geometrical influence on the acoustic properties.

### 2.2. Modal Analysis

**Figure 3:**
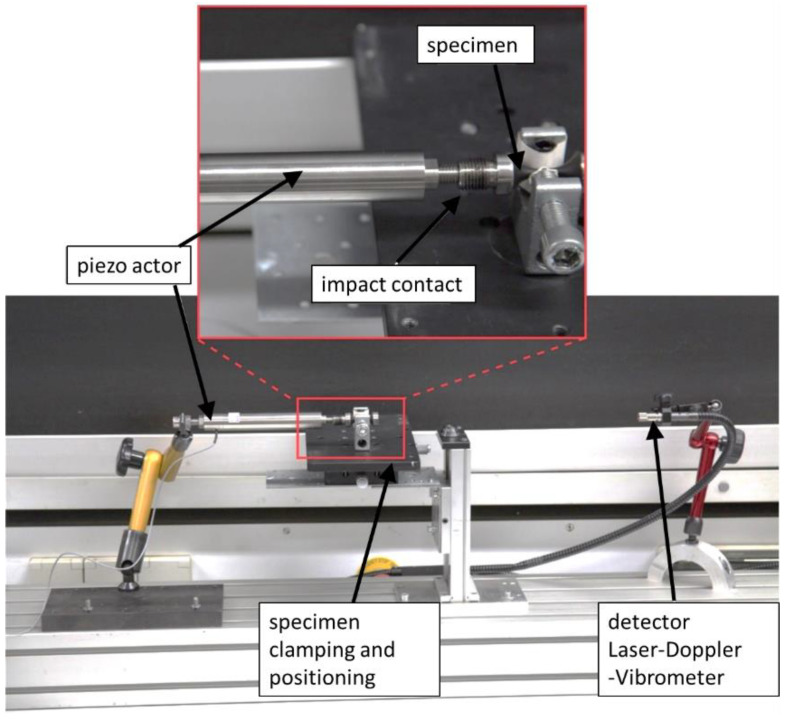
Experimental setup for modal analysis by LDV showing a VHCF-specimen.

**Figure 4:**
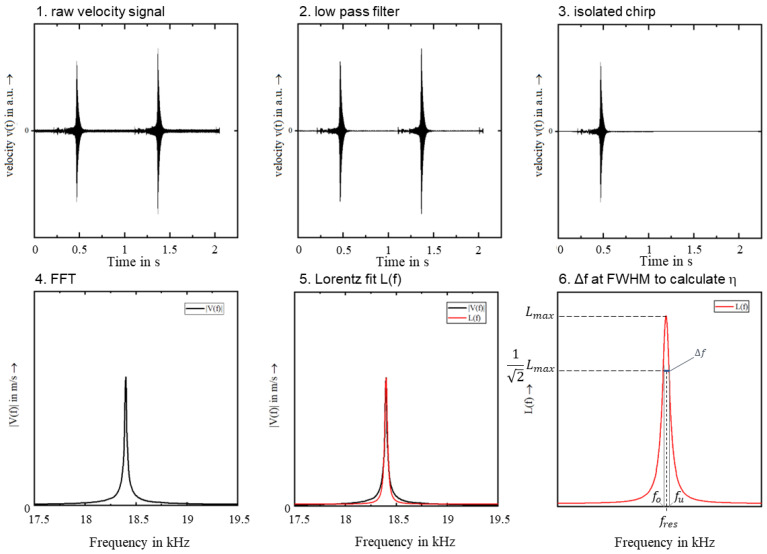
Schematic evaluation procedure for the measurement of the loss factor by FWHM-method chronologically from 1 to 6.

Acoustic properties were determined by modal analysis using Laser Doppler vibrometry (LDV: OFV-552, Polytec, Hörsching, Austria). The oscillation was induced by a piezo actor attached at the mounting surface, applying a frequency range from 10 kHz to 40 kHz. The digital signal generator of an oscilloscope (Picoscope 2205A, Pico Technology, Cambridgeshire, UK) generates a sinusoidal signal which first enters an amplifier (AKB-60, Monacor, Bremen, Germany) and then stimulates the piezo actor (P-844.60, PI, Karlsruhe, Germany) with a sufficient electrical voltage amplitude. The loose contact between specimen and actor allows one to measure solely the acoustic behaviour of the specimen. The resulting movement of the front surface of the specimen was detected by LDV. Fast Fourier Transformation (FFT) was used to determine the modal frequencies. The experimental setup for modal analysis by LDV is shown in Figure 3. A frequency spectrum indicating the modal frequencies, the acoustic fingerprint of the specimen, is the result of the modal analysis performed in this work as shown in Figure 4 and Figure 9.

The focus of investigations presented here is the loss coefficient η as a characteristic parameter for the detection of changes in the material or component, which can be used to predict approaching failure of components and to simplify future sonotrode development, by complementing the FE-Models used to determine the sonotrode geometry by more precise material properties. The procedure for determining loss coefficients in this work is the full width at half maximum (FWHM)-method based on the spectra gained by modal analysis. This evaluation method is often referred to as the −3 dB method or peak picking method [18]. A prerequisite for its applicability is that the measured system has only one degree of freedom or that the respective modes are sufficiently far apart on the frequency band [18]. The FWHM-method uses the shape and position of a resonance peak of the amplitude response of a forced oscillation to determine the loss coefficient. Ten measurements were performed at each specimen. Each individual measurement was evaluated separately, and the mean value of the loss coefficients was calculated. A small frequency gradient is referred as a sweep and larger frequency gradients as a chirp [19]. In the experiments carried out here, a chirp was used with the parameters given in Table 6.

At high frequencies, as considered here, it is recommended to measure the velocity instead of the displacement with the LDV, since at high frequencies and thus high velocities the displacements are usually very small [18,19]. The evaluation of the results is performed according to the schematic procedure shown in Figure 4.

After importing the velocity signal v(t) in the time domain (1), a low-pass filter with cut off frequency f_G_ = 40 kHz is applied over the raw data (2) to reduce high-frequency noise. Then a single continuous chirp with a duration of 0.9 s is isolated from the over 2 s long measurement signal (3). This part of the velocity signal is then transformed into the frequency domain by FFT and the amplitude is plotted in a diagram (4). At this point, ideally, an isolated resonance peak remains in the range of the natural frequency of the specimen, without being surrounded by closely spaced modes. The velocity amplitude in the frequency domain is then mapped by a suitable fit function (5). A Lorentz function was used for this purpose, since it turned out that the shape of the measured resonance peaks is particularly close to the shape of a Lorentz function according to (Equation (1)). The parameters of the curve—offset V_0_, centre f_Res_, width Δf and area A_L_—are determined iteratively. In addition, a weighting function was implemented which ensures that the maximum L_max_ is interpolated and the points near the maximum are particularly well approximated by the fit in Equation (1).

(1) $$L\left(f\right){=V}_{0}+\frac{{2\;\cdot \;A}_{L}}{\pi }\;\times \;\frac{w}{4\;{\left(\;f\;-{\;f}_{\mathrm{Res}}\;\right)}^{2}+w^{2}}$$

Knowing the Eigenfrequency f_Res_, the dimensionless loss coefficient can be determined using Equation (2).

(2) $$\eta =\frac{f_{o}-{\;f}_{u}}{f_{\mathrm{Res}}}$$

### 2.3. Very High Cycle Fatigue (VHCF) Experiments on AISI 347

Cyclic deformation induced phase transformation from paramagnetic austenite to ferromagnetic α′-martensite in AISI 347 was detected in specimens loaded in the VHCF regime by magnetic measurements using a Feritscope™ (FMP30, Helmut Fischer GmbH, Sindelfingen, Germany) [17]. Note that the linear relationship between the measured Feritscope™ unit in FE-% and the actual vol% of α′-martensite of the investigated material was determined systematically by XRD measurements and can be described as 1.57 × FE-% = vol% α′-martensite. However, in accordance with previous studies, the amount of α′-martensite is given in originally measured FE-%. During cyclic loading not only phase transformation occurs, but also further microstructural changes e.g., development of dislocation structures, stacking faults, twins and microcracks [4,5]. Opposed to the ξ value, that correlates only with the ferromagnetic fraction of α′-martensite, the determination of the loss coefficient offers the possibility for a non-destructive detection of the whole changes in the microstructure by cyclic loading.

**Figure 5:**
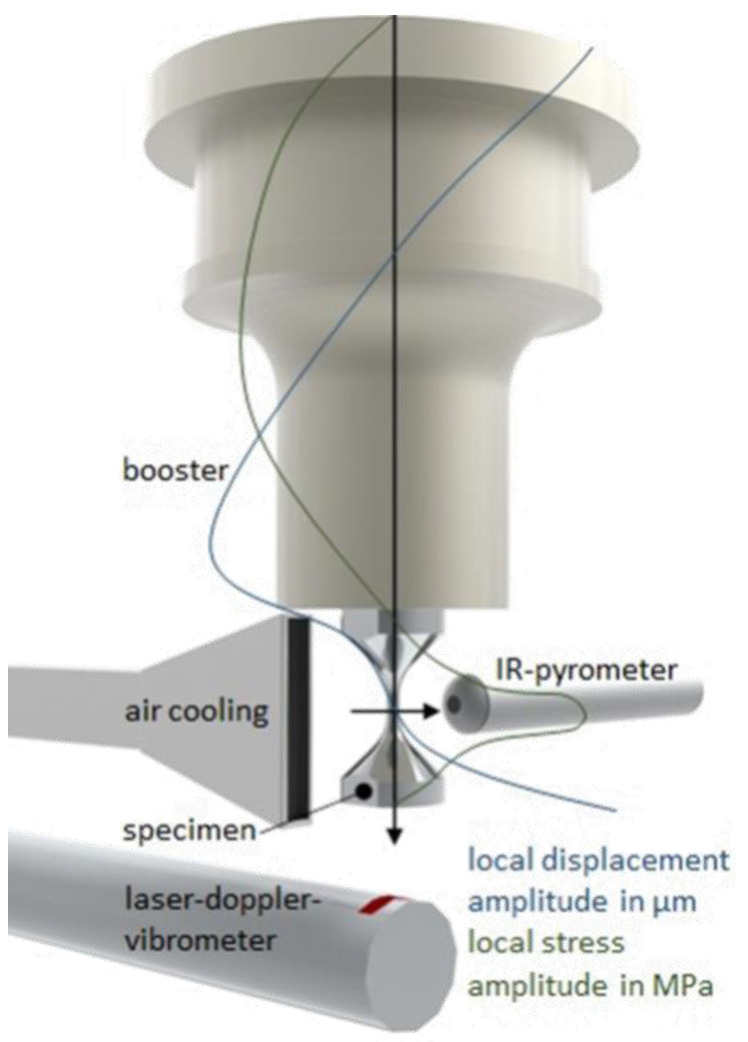
CAD sketch of an ultrasonic fatigue testing system including the local displacement amplitude and the local stress amplitude along the oscillation system, schematically.

The VHCF specimens were loaded and examined in several interrupted experiments with the aid of an ultrasonic fatigue testing (USFT) system. The USFT system operates at a frequency in the range of 19.5–20.5 kHz and is shown in Figure 5. Several additional subsystems are integrated, such as an LDV for measuring the displacement of the specimen, as well as a compressed air-cooling system and a pyrometer for non-contact temperature measurements.

The ultrasonic fatigue tests were performed in pulse-pause mode with push-pull loading (load ratio R = −1) at the displacement amplitude s_a_ = 9.5 μm, resulting in a stress amplitude σ_a_ = 250 MPa assuming linear elastic behaviour. The fatigue test started with 40 ms pulse and 3000 ms pause duration, which corresponds to an effective test frequency of f_eff,1_≈260 Hz. Since the metastable austenitic steel AISI 347 shows pronounced cyclic hardening caused by deformation induced α′-martensite formation, pulse-pause ratios of up to 60 ms/1800 ms become possible after sufficiently high cycle numbers [20]. Thus, the effective frequency can be increased to f_eff,2_ ≈ 390 Hz and eventually to f_eff,3_ = f_eff,4_ ≈ 650 Hz. To maintain a constant mode and displacement amplitude of the vibration under these circumstances, the generator voltage and the proportional and integral (PI) control parameters have to be adapted during the test [20,21]. After the measurement at N = 10^8^ cycles, the PI parameters were adjusted without changing the effective frequency (f_eff,3_ = f_eff,4_ ≈ 650 Hz).

Throughout the fatigue test, the displacement of the specimens is measured with the vibrometer “CLV-2534” in conjunction with the measuring head “VIB-A-530” (Polytec, Hörsching, Austria), and the displacement amplitude s_a_ is recorded for each pulse as an averaged quantity. Pyrometry is used to measure the temperature difference between the temperature at the start of the pulse and the maximum temperature (ΔT_pulse_) for each pulse. Moreover, a Feritscope™ is used to determine the evolution of the content of ferromagnetic α’-martensite phase during the ultrasonic fatigue loading.

### 2.4. Thermal Treatment of Sonotrode Materials

**Figure 6:**
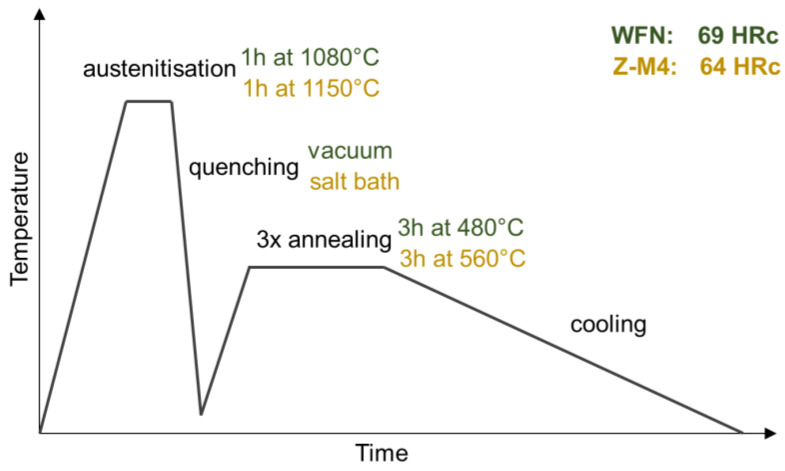
Thermal treatment of WFN and Z-M4 according to the data sheets [13,23].

To achieve the required hardness of sonotrode materials for ultrasonic welding, Z-M4 and WFN have to be thermally treated causing a thermally induced austenite-martensite phase transformation and resulting in an almost pure martensitic microstructure [14,22]. In principle the thermal treatment for Z-M4 and WFN is very similar as shown schematically in Figure 6. The austenitisation is followed by quenching and repeated annealing to finally achieve the requested hardnesses.

### 2.5. Determination of Young’s Moduli

Elastic moduli of the sonotrode materials were indirectly determined by Vickers micro indentation tests using a Fischerscope H100C S (Helmut Fischer GmbH, Sindelfingen, Germany) with a force of 1000 mN and 10 s holding time. Considering the load F, the indentation depth h and the area of the indentation projected onto the surface *A_c_*, the elastic modulus *E_e_* which is approximately equivalent to the Young’s modulus can be calculated by Equation (3) [24].

(3) $$E_{e}=\frac{\sqrt{\pi }}{2}\frac{1}{\sqrt{A_{c}}}\frac{dP}{dh}$$

The Young’s moduli of AISI 347 were determined by tensile test at specimen with 97 vol.% α′-martensite [25] and in solution annealed initial state, i.e., with a purely austenitic microstructure [15], presented previously. All determined values listed in Table 7 will be described uniformly as elastic modulus *E_e_* in the following text for an improved readability.

## 3. Results and Discussion

**Figure 7:**
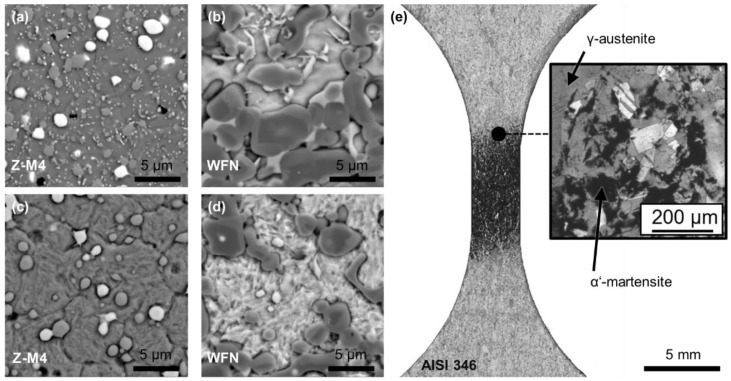
SEM backscattered electron (BSE) images of Z-M4 (**a**,**c**), WFN (**b**,**d**) before (**a**,**b**) and after martensite formation (**c**,**d**), and a light microscopic image of a AISI 346 VHCF-specimen after N = 10^9^ cycles at a stress amplitude of 270 MPa (**e**) [28].

The martensite formation was observed by SEM backscattered electron images in Z-M4 and WFN, and by light microscopy in AISI 346, respectively. Figure 7 shows the microstructure of the materials. The roundly shaped M_x_C (M = Cr, V, W, Mo) in Z-M4 [26,27] as well as TiC [14] in WFN are embedded in an initially smooth matrix (Figure 7a,b) that changes to a needle shaped phase, as it is characteristic for martensite (Figure 7c,d). Similarly, the microstructure of the longitudinal section of an AISI 346 VHCF-specimen after N = 10^9^ cycles at a stress amplitude 270 MPa is shown in Figure 7e. The specimen showed 0.00 FE-% in its shafts and 5.7 FE-% in the gauge length corresponding to the dark area that indicates the deformation induced α′-martensite and to the bright α′-martensite free areas in the shafts [28].

**Figure 8:**
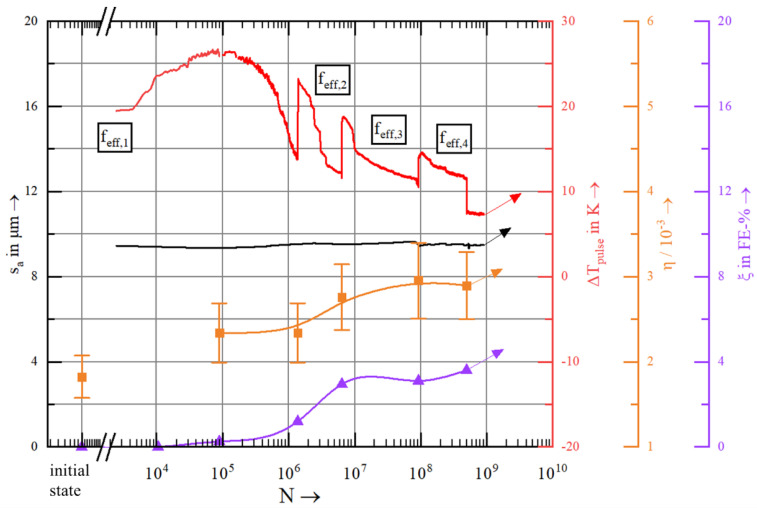
Development maximum displacement amplitude (s_a_), change in temperature during each load pulse (ΔT_pulse_) and the quasi in-situ measured magnetic phase fraction(ξ) as well as the loss coefficient factor (Ƞ) during ultrasonic fatigue testing with σ_a_ = 250 MPa at ambient temperature of AISI 347.

Figure 8 shows the displacement amplitude (s_a_), the temperature change within one pulse (ΔT_pulse_), the loss coefficient (η) and the content of ferromagnetic α′-martensite (ξ), which were determined during the test interruptions; they are plotted versus the number of load cycles N.

In the initial state, the measured ferromagnetic phase content of 0.00 FE-% verified the purely austenitic microstructure of AISI 347. At the beginning of the fatigue test, the pulse-pause ratio was set at 40 ms/3000 ms (f_eff,1_ ≈ 260 Hz) to keep the absolute specimen temperature below 50 °C despite the strong self-heating. ΔT_pulse_ increased until the first interruption of the experiment at N = 10^5^ cycles. At this point, a magnetic fraction of 0.25 FE-% was recorded, indicating the recent onset of deformation induced phase transformation from paramagnetic austenite to ferromagnetic α′-martensite.

When the specimen is further loaded to N = 10^6^ and N = 6 × 10^6^ cycles, the measured FE-% content increases continuously. In the same regime of cycle numbers, ΔT_pulse_ strongly decreases. Until N = 10^8^, the ferromagnetic phase content further increases to 3.5 FE-%, followed by a weaker increase until the test was terminated at N = 5 × 10^8^ cycles. The steps in the ΔT_pulse_ signal resulted from the increase of pulse time, which causes an increase in total heat generation during a single pulse. Nevertheless, ΔT_pulse_ continuously decreased after N = 10^5^, which indicates the cyclic hardening due to the reduction of strain energy density and correlates with stress-strain hysteresis loops [15].

**Figure 9:**
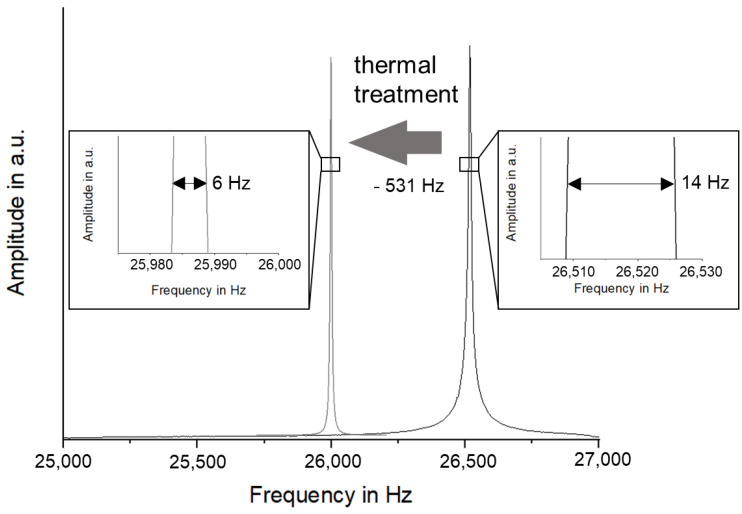
Changes in Eigenfrequency and loss coefficient of Z-M4 PM due to thermal treatment.

Initially, a loss coefficient of η = 1.83 × 10^−3^ was determined, which increased in the first test section up to N = 10^5^ cycles by 28.6%. Between N = 10^5^ and 10^6^ cycles, the loss factor remained constant. The subsequent, fourth measurement at N = 6 × 10^6^ cycles revealed a significant increase of the loss factor by 17.9%. In the same period between N = 10^6^ and N = 6 × 10^6^ cycles, the most pronounced α′-martensite formation was recorded. It can be seen from the trend line that deformation induced α′-martensite formation approaches a saturated state from N = 10^8^ cycles which roughly correlates with the evolution of the loss coefficient. As mentioned in the beginning, the sonotrode materials significantly change their acoustic properties due to thermal treatment. Most importantly, the Eigenfrequency changes by a couple of hundred Hz, which can make the sonotrode useless for application. A change of the grain shape and size by thermal treatment was not distinguishable in microstructural investigations, characteristic for metal-based sintered material [29]. The relatively large changes of the acoustic properties of a Z-M4 rod are shown in Figure 9.

In the case of lossless oscillation, the wavelength λ of any object depends on the Young’s modulus *E*, the mass density ρ and the Poisson number as given in Equation (4) for a rod shaped specimen [30].

(4) $$\lambda =\sqrt{\frac{E}{1-\nu }\frac{1}{\rho \left(1-\nu -2\nu^{2}\right)}}f_{calc}$$

Table 8 gives an overview of the changes in loss coefficient and Eigenfrequency determined by modal analysis as well as elastic moduli of all investigated materials.

The values of all listed properties are in the same magnitude for the considered materials, but in contrast to the sonotrode materials, AISI 347 shows an increasing loss coefficient. The Δ-values result from the values before and after thermal treatment for Z-M4 and WFN. For AISI 347 Δ and Δf were experimentally determined at N = 10^5^ and N = 5 × 10^8^ in this study. Δ*E* and f_calc_ that results from Equation (4) were calculated based on literature values [25].

Despite the large percentual change of the loss coefficients, the change of elastic moduli seem to be the main reason for the changes in Eigenfrequency, as the loss coefficients are too small to affect the Eigenfrequencies in the observed magnitude, shown by Equation (5) [30].

(5) $$\omega_{d}=\omega \sqrt{1-D^{2}}\;$$

The formation of martensite results in decreasing elastic moduli, suiting the values listed in Table 8 [25]. For the sonotrode materials (Z-M4 and WFN) the calculated change of the Eigenfrequency (Δf_calc_) based on the change of elastic modulus (Δ*E_e_* Equation (4)) fits the measured Eigenfrequencies (Δf) very well.

The loss coefficient of AISI 347 is expected to decrease due to the formation of α′-martensite [17]. However, an opposite behaviour is found in the presented data. Like previous studies (see Table 1. Additionally, grain size and shape that possibly change by thermal treatment, may affect the acoustic properties of the material [6,7].), the attenuation i.e., the loss coefficient can increase with raising load cycles. Cyclic hardening happens in AISI 347 that is accompanied by deformation induced α′-martensite formation as well as increasing density of dislocations and stacking faults [21]. Since an increase in the stacking fault density is a prerequisite for α′-martensite formation, the signals of loss coefficient and FE-% correspond qualitatively.

## 4. Conclusions

1. The determination of loss coefficient by FWHM-modal analysis was successfully used to notice changes in material properties for sonotrode materials (Z-M4 and WFN) by thermal treatment and in metastable austenitic stainless steel AISI 347 due to cyclic loading.
2. Although the loss coefficient was inappropriate to mathematically explain the change in microstructure and properties, a large difference before and after thermal treatment was recognized.
3. Changes in elastic moduli were identified to be most likely responsible for changes of the Eigenfrequency after thermal treatment of sonotrode materials, significantly simplifying future calculation and design of sonotrodes.
4. Investigations on AISI 347 showed, that loss coefficients strongly correlated to the content of fatigue induced ferromagnetic α′-martensite and hence, indicate microstructural changes accurately. However, the expected decrease of the loss coefficient due to the formation of α′-martensite was not confirmed, but rather a domination by other possible microstructural changes that were also shown in previous studies.
5. The hypothesis, that a determination of loss coefficients by non-destructive modal analysis, representing material changes, is suitable for quality control and process monitoring as the loss coefficient is very sensitive to small changes in materials and components, was confirmed.

## Figures and Tables

**Table 1:** Materials’ reactions on cyclic loading with respect to attenuation.

Material	Pure Copper	Steel AISI 1045 (S45C)	Aluminium Cast Alloy EN AC-42100	
**Loading**	Tensile	Circumferential bending	Circumferential bending	Tensile & compression
**Attenuation behaviour**	Peak of damping at 20–40% of the lifetime	Sudden peak at 70% of the lifetime	Sudden increase of attenuation	Continuous increase of attenuation until sudden decrease close to the fatigue failure
**Microstructural** **reactions**	Dislocation movement and formation of slip bands	Interaction of dislocations	Increasing dislocation density & cyclic hardening	Increasing dislocation density & cyclic hardening until saturation followed by crack initiation
**Source**	[8]	[9]	[3]	

**Table 2:** Chemical composition of Zapp Z-M4 PM in wt% [12].

Fe	C	Mn	Si	Cr	V	Mo	W
79.3%	1.4%	0.3%	0.3%	4%	4%	5.2%	5.5%

**Table 3:** Chemical composition of Ferrotitanit WFN in wt% [13].

Steel Matrix (67%)				Ceramic Phase (33%)
**Fe**	**C**	**Cr**	**Mo**	**TiC**
82.75%	0.75%	13.5%	3.0%	100%

**Table 4:** Chemical composition of AISI 347 in wt%.

Fe	C	N	Cr	Ni	Mn	Mo	Si	P	Cu	Nb
Bal.	0.04	0.007	17.60	10.64	1.83	0.29	0.41	0.02	0.06	0.62

**Table 5:** Grain and particle sizes of the investigated materials.

	Z-M4	WFN	AISI 347
Grain size in µm	1–3	0.3–3	120
Size of reinforcing phase in µm	1–2	5–8	-
Content of reinforcing phase in area%	20	33	-

**Table 6:** Measurement parameters for the modal analysis.

Frequency Gradient	Start Frequency	End Frequency	Measurement Time
1/3 × 10^5^ s^−2^	10 kHz	40 kHz	0.9 s

**Table 7:** Elastic moduli for austenitic and martensitic microstructures.

	Z-M4	WFN	AISI 347
*E_e_* austenite in GPa	250 ± 5	330 ± 7	183 [15]
*E_e_* thermally induced martensite in GPa	227 ± 5	305 ± 7	
*E_e_* deformation induced α′-martensite in GPa			175 [25]

**Table 8:** Changes in loss coefficient, elastic modulus and Eigenfrequency, of Z-M4, WFN and AISI 347 during the experiments.

	Z-M4	WFN	AISI 347
Δ*E_e_* in GPa	−26 ± 5	−23 ± 7	−8 [15,25]
Δ*E_e_* in %	−12	−6	−4
Δf in Hz	−700	−400	−100
Δf in %	−3.5	−2.0	−0.5
Δf_calc_ in % according to Equation (4)	−3.5	−2.4	−2.0
Δη × 10^−4^	−0.3	−2.8	+0.6
Δη in %	−57	−333	+20

## Data Availability

The data presented in this study are available on request from the corresponding author.

## References

[1] Liesegang M., Arweiler S., Beck T., Balle F. (2021). Orbital Ultrasonic Welding of Ti-Fittings to CFRP-Tubes. J. Manuf. Mater. Process..

[2] Liesegang M., Yu Y., Beck T., Balle F. (2021). Sonotrodes for Ultrasonic Welding of Titanium/CFRP-Joints—Materials Selection and Design. J. Manuf. Mater. Process..

[3] Giertler A., Krupp U., Michels W. (2011). Correlation of the fatigue damage with the damping behavior of a cast aluminum alloy. Mater. Test..

[4] Biermann H., Aneziris G.C. (2020). Austenitic TRIP/TWIP Steels and Steel-Zirconia Composites.

[5] Ghorbanhosseini S. (2020). A Review of the Failure and Damage Forms of Metals under Cyclic Loading. Int. J. Curr. Sci. Res. Rev..

[6] Ahn B., Lee S.S. (2000). Effect of microstructure of low carbon steels on ultrasonic attenuation. IEEE Trans. Ultrason. Ferroelectr. Freq. Control.

[7] Sha G., Huang M., Lowe M.J.S., Rokhlin S.I. (2020). Attenuation and velocity of elastic waves in polycrystals with generally anisotropic grains: Analytic and numerical modeling. J. Acoust. Soc. Am..

[8] Hirao M., Ogi H., Suzuki N., Ohtani T. (2000). Ultrasonic attenuation peak during fatigue of polycrystalline copper. Acta Mater..

[9] Ogi H., Hamaguchi T., Hirao M. (2000). Ultrasonic attenuation peak in steel and aluminum alloy during rotating bending fatigue. Met. Mat Trans A.

[10] Liesegang M., Beck T. (2021). Ultrasonic welding of magnetic hybrid material systems –316L stainless steel to Ni/Cu/Ni-coated Nd2Fe14B magnets. Funct. Compos. Mater.

[11] (2019). CPM 10V: Datasheet.

[12] (2019). Z-M4 PM: Datasheet.

[13] (2020). Ferrotitanit: Datasheet.

[14] Foller M., Meyer H. A new investigation on mechanical properties of ferro-titanit. Proceedings of the 6th International Tooling Conferece.

[15] Daniel T., Smaga M., Beck T. (2022). Cyclic deformation behavior of metastable austenitic stainless steel AISI 347 in the VHCF regime at ambient temperature and 300 °C. Int. J. Fatigue.

[16] Staab F., Liesegang M., Balle F. (2020). Local shear strength distribution of ultrasonically welded hybrid Aluminium to CFRP joints. Compos. Struct..

[17] Talonen J., Hänninen H. (2004). Damping properties of austenitic stainless steels containing strain-induced martensite. Met. Mat Trans A.

[18] He J., Fu Z.-F. (2001). Modal Analysis.

[19] Presas A., Valentin D., Egusquiza E., Valero C., Egusquiza M., Bossio M. (2017). Accurate Determination of the Frequency Response Function of Submerged and Confined Structures by Using PZT-Patches†. Sensors.

[20] Daniel T., Smaga M., Beck T., Schopf T., Stumpfrock L., Weihe S., Rudolph J. (2020). Investigation of the Very High Cycle Fatigue (VHCF) Behavior of Austenitic Stainless Steels and Their Welds for Reactor Internals at Ambient Temperature and 300 °C. Pressure Vessels and Piping Conference, Proceedings of the ASME 2020 Pressure Vessels & Piping Conference, Virtual, Online, 3 August 2020.

[21] Smaga M., Boemke A., Daniel T., Skorupski R., Sorich A., Beck T. (2019). Fatigue Behavior of Metastable Austenitic Stainless Steels in LCF, HCF and VHCF Regimes at Ambient and Elevated Temperatures. Metals.

[22] Briki J., Ben Slima S. (2008). A New Continuous Cooling Transformation Diagram for AISI M4 High-Speed Tool Steel. J. Mater. Eng. Perform..

[23] (2017). CPM RexM4: Datasheet.

[24] Franco A.R., Pintaúde G., Sinatora A., Pinedo C.E., Tschiptschin A.P. (2004). The use of a vickers indenter in depth sensing indentation for measuring elastic modulus and vickers hardness. Mater. Res..

[25] Skorupski R., Smaga M., Eifler D., Schmitt R., Müller R. (2013). Influence of Morphology of Deformation Induced α’-Martensite on Stress-Strain Response in a Two Phase Austenitic-Martensitic-Steel. Key Eng. Mater..

[26] Serna M.M., Rossi J.L. (2009). MC complex carbide in AISI M2 high-speed steel. Mater. Lett..

[27] Frisk K., Bratberg J., Markström A. (2005). Thermodynamic modelling of the M6C carbide in cemented carbides and high-speed steel. Calphad.

[28] Smaga M., Daniel T., Regitz E., Beck T., Schopf T., Veile G., Weihe S., Rudolph J., Fischer U. Very high cycle fatigue (VHCF) behavior of austenitic stainless steels and their welds for reactor internals at ambient and operating relevant temperatures. Proceedings of the 26th International Conference on Structural Mechanics in Reactor Technology.

[29] Danninger H., Dlapka M. (2018). Heat Treatment of Sintered Steels—What is different?. HTM J. Heat Treat. Mater..

[30] Möser M. (2009). Engineering Acoustics: An Introduction to Noise Control.

